# A multicenter observational study on Medication-Related Osteonecrosis of the Jaw (MRONJ) in advanced cancer and myeloma patients of a cancer network in North-Western Italy

**DOI:** 10.4317/medoral.24318

**Published:** 2020-12-19

**Authors:** Vittorio Fusco, Marco Cabras, Francesco Erovigni, Alessandro Dell’Acqua, Paolo Giacomo Arduino, Monica Pentenero, Paolo Appendino, Lorenzo Basano, Francesco Della Ferrera, Antonella Fasciolo, Majlinda Caka, Mario Migliario, Stefano Franchi, Alessio Gambino

**Affiliations:** 1 Oncology Unit, Azienda Ospedaliera di Alessandria, Italy; 2 Department of Surgical Sciences, Cir Dental School and University of Turin, Italy; 3 Department of Oncology, Oral Medicine and Oral Oncology Unit, San Luigi Gonzaga Hospital, University of Turin, Italy; 4 Department of Dentistry and Oral Surgery, Mauriziano Hospital, Turin, Italy; 5 Maxillofacial Surgery Unit, Alessandria Hospital, Alessandria, Italy; 6 Maxillofacial Surgery Unit, Asti Hospital, Italy; 7 Dental Clinic, Department of Health Science, University of Eastern Piedmont “A. Avogadro”, Novara, Italy; 8 Maxillofacial Surgery, Novara Hospital, Italy

## Abstract

**Background:**

Incidence of Medication-Related Osteonecrosis of the Jaw (MRONJ) related to cancer and myeloma treatments is undetermined, with scarce data varying from 2 to 7.8/million/year in limited investigated populations. A 9-years [2009-2018] regional-wide survey was conducted, deploying the North-Western Italy Cancer Network (“Rete Oncologica Piemonte e Valle d’Aosta”), to assess number and main characteristics of MRONJ cases among myeloma/cancer patients, within a population of 4.5 million inhabitants.

**Material and Methods:**

MRONJ cases were collected retrospectively from January 2009 to June 2015; from July 2015 to December 2018, data were collected prospectively. Number of new MRONJ cases per year, underlying disorder, drug(s) administered, treatment duration, site and onset timing of MRONJ were detailed.

**Results:**

459 MRONJ cases were identified. Primary diseases were breast cancer (46%), prostate cancer (21%), myeloma (19%), and other types of carcinoma (14%). Patients received antiresorptive treatment either alone (399; 88.47%) or in combination with biological agents (52; 11.53%); 8 patients (1.7%) received only antiangiogenic drugs. Zoledronic acid [388] and denosumab [59] were the most frequently administered drugs. Mandible was involved in 296 (64,5%) cases. Number of new MRONJ cases was stable from 2009 to 2015, with a mean of 51.3 cases per year (raw incidence: 11.6/million/year), declining in the 2016-2018 years to 33.3 cases per year (raw incidence: 7.5/million/year).

**Conclusions:**

With such discrepancy of cases overtime being partially explicable, number of new MRONJ cases per year are consistent with those observed in a previous study [2003-2008] in the same region, being instead higher than those reported in other populations.

** Key words:**Osteonecrosis of the jaw, bisphosphonates, zoledronic acid, denosumab, antiangiogenic drug, incidence.

## Introduction

Osteonecrosis of the jaw (ONJ) in patients receiving antiresorptive drugs (bisphosphonates, denosumab) – also known as Bone Modifying Agents (BMA) - or other drugs is now referred also as MRONJ (Medication-Related Osteonecrosis of the jaw) (1). MRONJ is a relatively new disease, recognized only since 2003, potentially affecting the quality of life of cancer and myeloma patients (2). A largely adopted definition (with accompanying staging system) based on an 8-week observation of bone exposure in oral cavity was confirmed in the latest AAOMS position-paper (1), but the presence of cases without bone exposure made that definition controversial (3,4).

The real incidence and prevalence of MRONJ in general population are actually unknown, as epidemiology data is scarce due to all a series of problematic coding of the disease (5,6). Few population-based studies using clinical data from patient charts of all the observed cases have been published (7-11), as collection of clinically confirmed MRONJ cases outside of clinical trial settings is difficult (6). Attempts were conducted with insurance database or large national healthcare system database, but even specific algorithms showed important limitations or inconsistent results (12,13). Results from a large Scandinavian registry of all clinically confirmed MRONJ cases observed in three nations are awaited with great interest (6,14).

A previous attempt to ascertain epidemiological data in North-Western Italy was conducted up to 2008 collecting a relatively high number of ONJ cases (200 subjects in a limited number of years) in cancer and myeloma patients in a 4.3 million population, by the clinical charts of a regional Cancer Network (15).

The scenario of observed MRONJ cases and of drug prescriptions in cancer and myeloma patients changed afterwards. A reduction of long-term prescription habits of BMAs (4) and the introduction of monthly denosumab as an alternative to monthly zoledronic acid (and other intravenous bisphosphonates) (16) might have impacted on the incidence of MRONJ cases in real world (17). More recently, delayed zoledronic acid infusions (every 12 weeks instead of every 4 weeks) has been proposed (18) but the impact of this schedule in clinical setting is not yet known.

New data of clinically confirmed MRONJ cases in cancer and myeloma patients of Rete Oncologica Piemonte e Valle d’Aosta have been collected in years 2009-2018 for updated evaluations.

## Material and Methods

In June 2015, the regional ONJ Study Group and Supportive Care Study Group established by members of the Piedmont and Valle d’Aosta cancer network (Rete Oncologica) in North-Western Italy launched a survey to retrospectively collect data of MRONJ cases, diagnosed after January 1st 2009, at each center of the network.

A case data collection form was sent to each Oncology and Hematology Unit of the Network and to all the referral oral care centers (Maxillofacial Surgery; Oral Medicine and Oral Surgery departments) in the region.

A collection of cases was conducted after cross-checking reports from medical oncology, hematology, and oral care centers, in order to avoid double counting. Only forms with sufficient data were then included in an appropriate database.

Furthermore, since 1st July2015, new suspected and recognized MRONJ cases were prospectively collected from six of the aforementioned oral care centers and evaluated up to 31st December 2018.

Data collection concerning cancer history and occurrence of MRONJ was further refined by two Authors who visited Oncology/Hematology units, and Maxillofacial or Oral Care units, respectively.

MRONJ cases with bone exposure, and no history of radiation therapy according to the definitions of American Association of Oral and Maxillofacial Surgeons (AAOMS) (1), as well as cases without frank bone exposure according to SIPMO-SICMF clinical and radiological criteria (19) were collected.

The following data were registered and analyzed:

(a) demographics: age, sex;

(b) cancer history: type of solid primary cancer, or myeloma;

(c) antiresorptive/antiangiogenic therapy: supposed triggering drug (or sequence), duration of therapy;

(d) site (maxillary versus mandible or both);

(e) year of MRONJ diagnosis;

Median (interquartile range, IQR) or mean (Standard Deviation, SD) were applied to continuous data, whilst numbers (percentage, %) have been applied for categoric data. Analyses were carried out with version 15.1 of STATA SE (STATA Corp, USA).

## Results

Between January 2009 and December 2018, we collected data of 603 cases of MRONJ. Of these, 144 developed MRONJ due to antiresorptive treatment of bone disease (osteoporosis, osteopenia, Paget’s disease, etc) other than cancer metastatic to bone or myeloma. These cases were excluded, going beyond the scope of this paper.

- Patients’ characteristics

The main characteristics of the remaining 459 MRONJ cases in cancer and myeloma patients are presented in Table 1. Females amounted to 63% of the entire sample. Breast cancer was the most represented disease (211 cases, 46%), followed by prostate cancer (94 cases, 21%) myeloma (89 cases, 19%), and other types of carcinoma (65 cases, 14%).

**Table 1:**
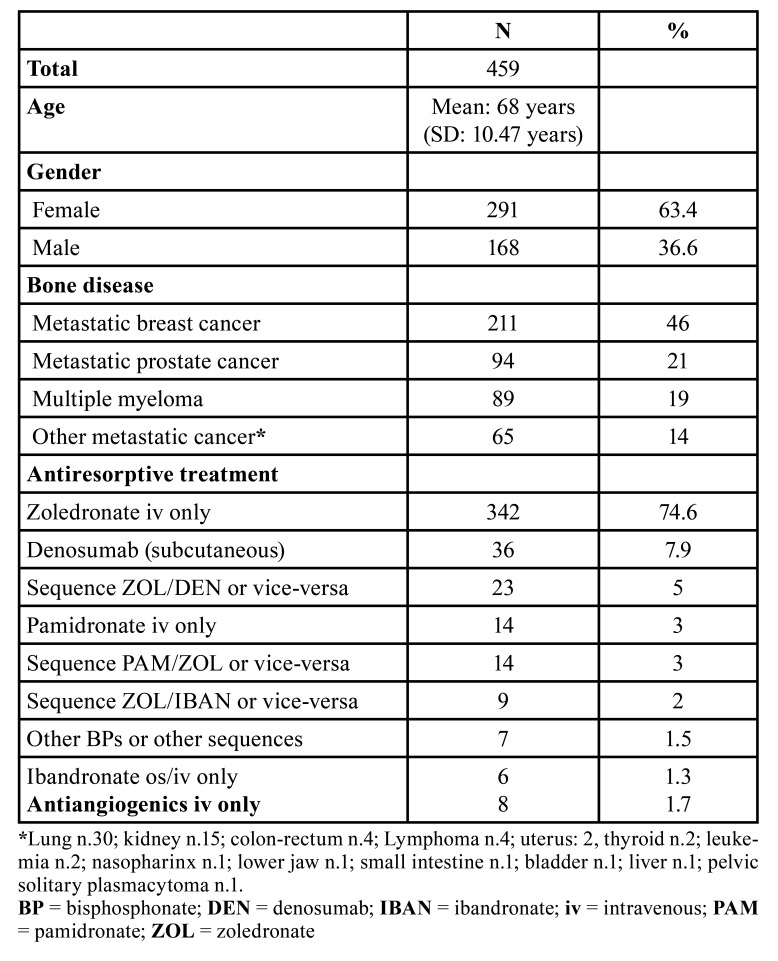
Main characteristics of MRONJ patients.

- Drug and duration of therapy

Overall, 451 cases of MRONJ received antiresorptive drugs (bisphosphonates, denosumab): 399 (88.47%) underwent treatment with either one antiresorptive alone or as sequence of antiresorptive agents (and no antiangiogenic drug), while the remaining 52 (11.53%) were administered with either one antiresorptive agent or as sequence of antiresorptive agents in combination with biological agents.

Eight cases of MRONJ (1.7%) were detected on patients under antiangiogenics without history of antiresorptive treatment (4 bevacizumab, 4 sunitinib).

Zoledronic acid was by far the most frequently administered drug (388 cases globally), with 342 (74.6%) patients undergoing this treatment as single antiresorptive drug. Denosumab was the sole therapeutic option in 36 (7.9%) cases, whereas 23 (5%) cases where exposed to both denosumab and zoledronic acid. Other single antiresorptive drugs (pamidronate, ibandronate, etc.) or drug sequences were less frequently registered (Table 1).

Out of 52 patients undergoing antiresorptive drugs, either alone or as a sequence, with biological agents, 12 received thalidomide, 11 lenalidomide, 11 bevacizumab, 9 everolimus, and 9 sunitinib.

**Figure 1:**
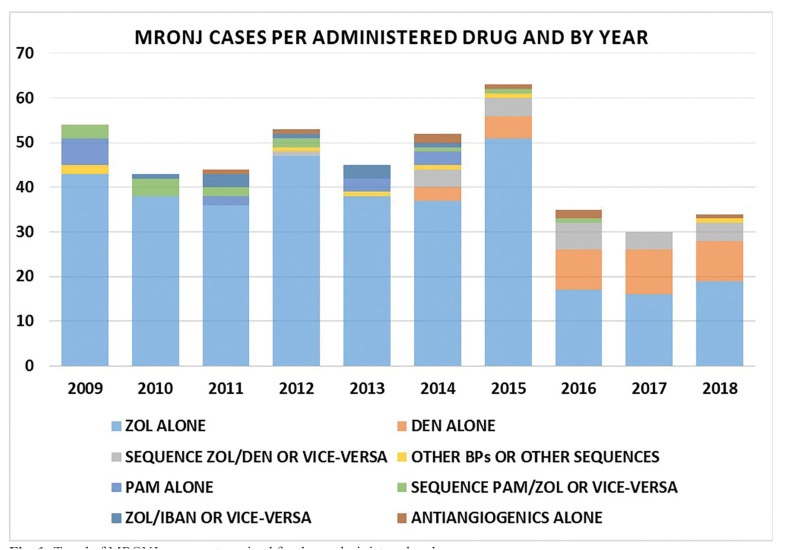
Trend of MRONJ cases, categorized for drug administered and year.

Table 2 and Fig. 1 show the distribution of MRONJ diagnosis per year, according to the main agent/sequence of drugs administered.

Table 3 illustrates the median duration of antiresorptive/antiangiogenic therapy.

- Clinical manifestation

The majority of patients (296; 64,5%) had a lesion in the mandible; in 125 (27.2%) patients MRONJ lesions occurred in the maxilla, whereas in 38 (8.3%) patients MRONJ occurred in both maxilla and mandible.

- Number of ONJ cases by period of data collection

**Figure 2:**
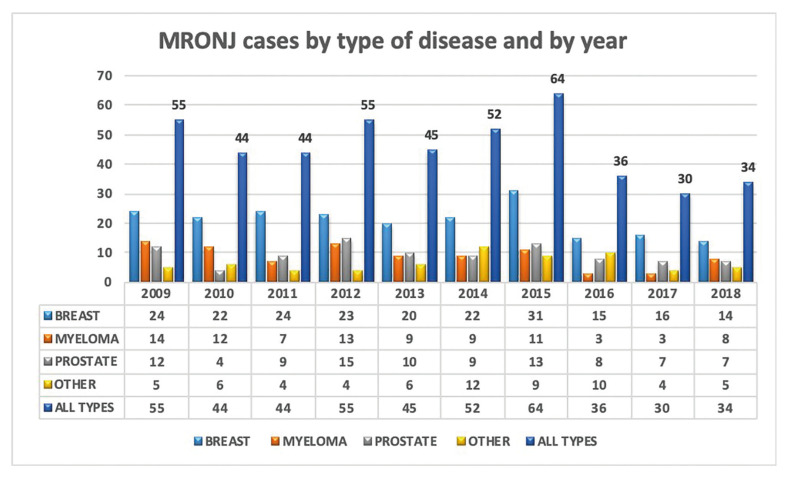
Number of MRONJ cases by type of disease and by year.

The pattern of distribution of MRONJ cases over time is depicted in Fig. 2. It showed steady incidence of MRONJ per year from 2009 to 2015 of retrospective data collection, with a mean of 51.3 cases per year, and a raw unadjusted incidence of 11.6 MRONJ cases/million/year. Conversely, during the 2016-2018 period, when data were collected prospectively from the six main collaborating oral care centers, we observed a mean of 33.3 cases per year, with a raw unadjusted incidence of 7.5 MRONJ cases/million/year.

**Table 2:**
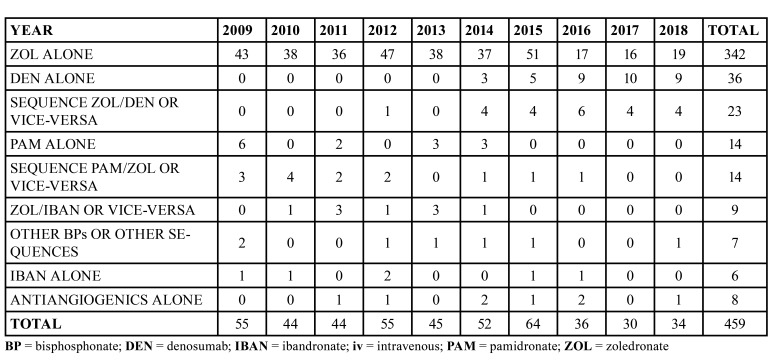
MRONJ cases, categorized for drug administered and year.

**Table 3:**
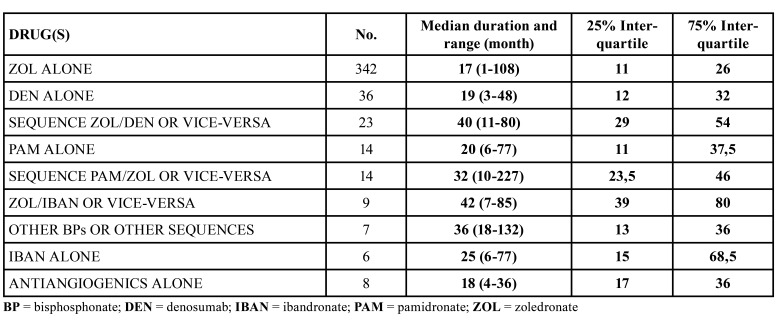
Duration and dose of antiresorptive/antiangiogenic treatment at the MRONJ onset time.

## Discussion

Incidence and prevalence of MRONJ in cancer and myeloma patients exposed to antiresorptive and/or antiangiogenic drugs are unknown. Accurate studies are not yet available in literature, due to a series of issues, including: heterogeneity and challenging accountability of the exposed population; uncertainty of diagnosis and adjudication of MRONJ disease; variability of clinical data collection of suspected and ascertained cases; eventual time changing of the therapeutic protocols in cancer patients; discrepancy between sources of data (for example reporting to Drug Safety Pharmacovigilance agencies versus clinical surveys and multicenter studies) (5,6).

Consequently, large case collection in defined populations are needed. While collection of cases on survey invitation basis may be incomplete or insufficient due to uncertainty of investigated population (20,21), studies based on population-based systematic registration of all clinically confirmed MRONJ cases seem more useful to increase understanding and to improve patient management (6). To the best of our knowledge, few studies have reported reliable data, useful for evaluation of MRONJ incidence, either through surveys or through cohort studies, on a regional and/or nationwide scale. Of these, it is worth mentioning the early efforts of Mavrokokki *et al* (7), who conducted a 2-year [2004-2005] nationwide postal survey among oral and maxillofacial surgeons in Australia, recollecting 82 MRONJ among cancer/myeloma patients. With a population at the time of 20.3 million people, a raw unadjusted incidence of about 2 MRONJ cases/million/year could be inferred. More recently, epidemiologic endeavors in Scandinavian regions lead to higher incidence of MRONJ in myeloma/cancer patients. Kruger *et al*. (8) reported 103 MRONJ cases after intravenous bisphosphonates in a 8-year [2003-2010] survey conducted in Norway, whose population at that time was 5.3 million population, leading to a raw incidence of circa 2.4 MRONJ cases/million/year. Hallmer *et al* (9) conducted a four-year [2012-2015] prospective cohort study in the Swedish region of Skåne, whose population was nearly 1.3 million, in which 24 MRONJ cases amid cancer and myeloma patients were detected, leading to a raw incidence of 4.6 MRONJ cases/million/year. Furthermore, Corrraini *et al* (10) registered 175 MRONJ cases in a 4-year [2012-2015] prospective nationwide cohort study in Denmark; with a population of 5.6 million at that time, a raw incidence of 7.8 MRONJ cases/million/year can be assessed.

In our previous experience (11) as a 5-year [2003-2008] regional study group within the Piedmont and Valle d’Aosta network (Rete Oncologica), we collected MRONJ cases among cancer patients from a population of 4.3 million of inhabitants. Briefly, 58-60 cases per year were detected during 2005 and 2006, with a peak incidence of more than 13 cases/million/year, followed by a decrease in the subsequent two years, with 37 cases in 2007, and 21 cases in 2008, decreasing its incidence to respectively 8.6 and 4.8 cases/million/year in last two observation years. Once again, these estimates must be interpreted as a raw unadjusted incidence. In any case, this trend paralleled that of prescriptions of intravenous bisphosphonates in Italy (11).

A similar decrease tendency was observed in Australia, being there mainly attributed to the implementation of “preventive” measures (22).

Going to analyze data of years after 2008, we expected a similar trend in the following years, based on the hypothesis that MRONJ occurrence would decrease due to both the efficacy of measures adopted by clinicians and oral specialists to reduce MRONJ risk, and due to the reduction of overall bisphosphonate prescriptions, as shorter treatment duration were more recently recommended (4,16). However, the present data collection showed a substantially unchanged number of new MRONJ cases per year from 2009 to 2015 in our cancer patient population in comparison with the previous period of observation, with a mean of 51.3 (range 44-64) cases per year, and a raw unadjusted incidence of 11.6 cases/million/year, next to the peak incidence of years 2005-2006.

The present study confirms that MRONJ is not a rare adverse event in at risk cancer patients in North-Western Italy, with 459 cases observed in latest 10-year timespan and a peak of 64 cases in 2015, according to our experience. On this basis, we might infer an estimation of up to 930 new MRONJ cases per year to be diagnosed on a national basis, throughout Italy.

Furthermore, data presented in this work must take into account a change in drug prescription habits in cancer and myeloma patients: differently from the partial substitution of pamidronate with zoledronic acid during the 2003-2008 timespan registered in our previous work (11), since 2013 we observed the introduction of denosumab for solid cancer Italian population, at monthly 120 mg subcutaneous injections. Denosumab was administered either as a “switch drug” for patients already undergoing treatment with bisphosphonates (mostly zoledronic acid), or as first-line antiresorptive treatment, as observed in other countries (23,24). Consequently, denosumab-related MRONJ cases have been registered in our cohort since 2013, with 36 cases from patients undergoing treatment with denosumab alone, and 23 cases in patients switching either from zoledronic acid to denosumab, or vice-versa.

Regarding the median latency between the start of therapy and onset of MRONJ (Table 3), no significant difference was found among the main therapy groups. The interquartile evaluation revealed that most of MRONJ cases were observed between the end of the first year and the third year of treatment. However, the wide range observed among all the treatment groups implies that each single patient might be at risk of MRONJ already after few months of therapy, as well as after many years, with cases of MRONJ diagnosed after 108 up to 227 months (of treatment or follow-up) since the start of antiresorptive treatment. Actually, MRONJ patients who received more than one antiresorptive drug had generally a more delayed disease onset, as they were medially exposed to higher cumulative drug dose, and experienced a longer survival: this clearly seems a selection bias and this chain of events should be evaluated by controlled trials.

The main strength of the present work relies on the long-term duration of the observation and data collection, conducted for almost a decade in the restricted territory of Piedmont and Valle d’Aosta with a relatively well defined population, leading us to believe that most - if not all – MRONJ cases occurring between 2009 and 2018 have been included, as well as in the previous 2003-2008 experience (11),

On the other hand, one limit of the study relies on the heterogeneity of data recollection, with data from 2009 to June 2015 acquired retrospectively, and data acquired prospectively since mid-2015. Anyway, prospective data collection was based on cases from the six main and larger oral care centers, in which 98% of MRONJ cases occurring between 2009 and 2015 were registered. However, such difference in data collection can only partially explain the apparent reduction in the number of MRONJ cases observed from 2016 to 2018 in comparison with 2009-2015 timeline; this result needs further observation time.

Another limit of our study, as in previous studies, is the impossibility to obtain a properly adjusted incidence and prevalence of MRONJ among “at risk” cancer/myeloma patients in our territory, due to the lack of a precise estimate of the total of patients undergoing each one of the aforementioned treatments (bisphosphonates, denosumab, antiangiogenic drugs) in Piedmont-Valle d’Aosta. We are attempting to overcome this obstacle by launching a research in the regional hospital-based pharmacy digital database, and thus determining the actual number of antiresorptive drug prescriptions; we are investigating the use Anatomical Therapeutic Chemical (ATC) codes for pamidronate, zoledronic acid and denosumab, while excluding formulations usually prescribed to non-cancer patients (i.e. zoledronic acid 5 mg, and denosumab 60 mg).

In summary, the present series aligns with the findings of our previous paper (11) and recent literature (7-13,25-27): MRONJ is not a rare adverse event in cancer and myeloma patients, and should require continuous attention and awareness by prescribers (oncologists, hematologists), oral and maxillofacial specialists, dental practitioners, as well as health policy stakeholders.
